# Factors Associated With State-Specific Medicaid Expansion and Receipt of Autologous Breast Reconstruction Among Patients Undergoing Mastectomy

**DOI:** 10.1001/jamanetworkopen.2021.19141

**Published:** 2021-08-03

**Authors:** Kristine A. Huynh, Mayank Jayaram, Chang Wang, Megan Lane, Lu Wang, Adeyiza O. Momoh, Kevin C. Chung

**Affiliations:** 1 Oakland University William Beaumont School of Medicine, Rochester, Michigan; 2 Section of Plastic Surgery, Department of Surgery, University of Michigan Medical School, Ann Arbor; 3 Department of Biostatistics, University of Michigan, Ann Arbor

## Abstract

**Importance:**

Despite demonstrated psychosocial benefits, autologous breast reconstruction remains underutilized. An analysis of the association between Medicaid expansion and autologous breast reconstruction has yet to be performed.

**Objective:**

To compare autologous breast reconstruction rates and determine the association between Medicaid expansion and breast reconstruction.

**Design, Setting, and Participants:**

A retrospective cross-sectional study was performed using the State Inpatient Database from January 1, 2012, through September 30, 2015, and included 51 340 patients. Patients were identified using the *International Classification of Diseases, Ninth Revision*, codes for breast cancer, mastectomy, and autologous breast reconstruction. Data from states that expanded Medicaid (New Jersey, New York, and Washington) were compared with states that did not expand Medicaid (Florida, North Carolina, and Wisconsin). Data were analyzed from June 1, 2020, through February 28, 2021.

**Exposures:**

The Patient Protection and Affordable Care Act’s Medicaid expansion was implemented in 2014; the preexpansion period ranged from 2012 to 2013 (2 years), whereas the postexpansion period ranged from 2014 to 2015 quarter 3 (1.75 years).

**Main Outcomes and Measures:**

Primary outcomes included use of autologous breast reconstruction before and after expansion. Independent covariates included patient demographics, comorbidities, and state of residence.

**Results:**

Among 45 850 patients who underwent mastectomy and 9215 patients who received autologous breast reconstruction, 36 777 (67%) were White and 32 205 (59%) had private insurance. The use of immediate or delayed autologous reconstruction increased from 18.1% (4951 of 27 290) to 23.0% (4264 of 18 560) throughout the study period. Compared with 2012, the odds of reconstruction were 64% higher in 2015 (odds ratio [OR], 1.64; 95% CI, 1.48-1.80; *P* < .001). African American (OR, 1.43; 95% CI, 1.33-1.55; *P* < .001) and Hispanic (OR, 1.44; 95% CI, 1.31-1.60; *P* < .001) patients had higher odds of reconstruction compared with White patients regardless of state of residence. However, Medicaid expansion was associated with a 28% decrease in the odds of reconstruction (OR, 0.72; 95% CI, 0.61-0.87; *P* < .001) for African American patients, a 40% decrease (OR, 0.60; 95% CI, 0.50-0.74; *P* < .001) for Hispanic patients, and 20% decrease (OR, 0.80; 95% CI, 0.67-0.96; *P* = .01) for patients with Asian, Native American, or other minority race/ethnicity. Medicaid expansion was not associated with changes in the odds of reconstruction for White patients.

**Conclusions and Relevance:**

In this cross-sectional study, although the odds of receiving autologous breast reconstruction increased annually, Medicaid expansion was associated with decreased odds of reconstruction for African American patients, Hispanic patients, and other patients of color.

## Key Points

**Question:**

What is the association between the Patient Protection and Affordable Care Act’s state-specific Medicaid expansion and the use of autologous breast reconstruction?

**Findings:**

In this national cross-sectional study of 45 850 patients who underwent mastectomy and 9215 patients who received autologous breast reconstruction, although the proportion of patients undergoing autologous breast reconstruction increased from 18.1% to 23.0% over the study period, Medicaid expansion was associated with significantly decreased odds of undergoing reconstruction for African American, Hispanic, Asian, and Native American patients.

**Meaning:**

Medicaid expansion was associated with decreased odds of autologous reconstruction for African American, Hispanic, and other patients with minority race/ethnicity.

## Teaser

This cross-sectional study investigates the association between Medicaid expansion and autologous breast reconstruction rates among women who underwent mastectomy.

## Introduction

Nearly 1 in 8 women in the United States will be diagnosed with breast cancer, and approximately one-third of these women will undergo mastectomy as treatment.^1,2,3^ Despite the well-known benefits of breast reconstruction such as improved self-esteem, enhanced body image, and decreased anxiety levels,^4,5,6^ approximately 40% of patients do not pursue reconstructive options in the US.^7^ Breast reconstruction includes both implant-based operations as well as autologous procedures, for which a patient’s muscle or soft tissue is used to reconstruct a breast. Several health care policies have been implemented to increase use of postmastectomy breast reconstruction (PBR). The Women’s Health and Cancer Rights Act (WHCRA) of 1998 aimed to mitigate financial barriers by requiring all health insurance companies that covered mastectomies to also provide coverage for breast reconstruction.^8^ Three years later, Congress passed additional legislation that enforced penalties against insurers that did not comply with WHCRA guidelines.^9^ In 2001, New York expanded their Medicaid program to increase insurance coverage for low-income families, which increased surgical volume across surgical subspecialties, including breast reconstruction.^10^ However, there was a disproportionate increase in implant-based reconstruction compared with autologous-based methods.^10,11^ The rates of PBR have increased by an average of 20% since 1998.^7^ However, this increase is widely variable based on geographic location; recent studies have demonstrated that disparities still persist based on an individual’s age, educational level, insurance type, race/ethnicity, and income.^12,13^

In 2014, 35 states underwent Medicaid expansion under the Patient Protection and Affordable Care Act (ACA) to increase health insurance coverage for individuals living at or below 138% of the Federal Poverty Level.^14^ The ACA aimed to improve timely access to care, thereby reducing late presentation and enhancing health outcomes.^15^ More than 20 million US residents have gained health insurance coverage through Medicaid expansion by 2016.^15,16^ Within the first year, women living in states that underwent expansion were more likely to receive recommended breast and cervical cancer screening, as well as seek more timely care for various surgical pathologies and cancers.^17,18,19,20^

Similar to other complex disease processes, Medicaid expansion has affected the delivery of breast cancer care and breast reconstruction. Health care legislation has increased the overall use of implant-based breast reconstruction.^21,22^ However, autologous breast reconstruction continues to be underused despite better psychosocial well-being and satisfaction. The association between 2014 Medicaid expansion and the use of autologous breast reconstruction remains unclear. In this study, we applied difference-in-differences technique to identify disparities that may exist in access to autologous breast reconstruction between expansion and nonexpansion states. Results from this study may provide insights into the association between the 2014 Medicaid expansion and the use of specialized care such as breast reconstruction and guide future health policy legislation.

## Methods

### Data Source

We used the State Inpatient Database from the Healthcare Cost and Utilization Project for this analysis. The State Input Database encompassed approximately 97% of US community hospital discharges and permits for highly reliable state-level comparisons of patient care within hospitals.^23^ The University of Michigan institutional review board deemed this study was exempt from review and did not require informed patient consent because this study used a large deidentified claims database. The authors followed the Strengthening the Reporting of Observational Studies in Epidemiology (STROBE) reporting guideline.^24^

### Study Population

The study cohort consisted of 3 expansion states (New York, Washington, and New Jersey) and 3 nonexpansion states (Florida, North Carolina, and Wisconsin) from geographically diverse regions across the country. The preintervention period included data from January 2012 to December 2013 (2 years), and the postintervention period included data from January 2014 through the third quarter of 2015 (1.75 years). Data from the fourth quarter of 2015 and beyond were excluded to minimize errors in documentation as health care professionals transitioned to the new *International Classification of Disease, Tenth Revision (ICD-10)* diagnosis and procedure codes.^25^

For this analysis, we included all female patients with breast cancer who underwent mastectomy between the ages of 18 and 90. *International Classification of Disease, Ninth Revision (ICD-9)* diagnostic and procedure codes^26^ were used to identify females with breast cancer, patients who received mastectomies, and patients who underwent immediate or delayed autologous breast reconstruction following mastectomy treatment (eTable 1 and eTable 2 in the Supplement). Patients who had implant-based reconstruction or secondary malignant neoplasms of the breast and skin were excluded from the study cohort. Additionally, individuals with missing data related to our variables of interest were excluded from the analysis (eFigure 1 and eTable 3 in the Supplement).

### Outcomes and Variables of Interest

The primary outcome of interest was use of immediate or delayed autologous breast reconstruction following mastectomy pre– and post–Medicaid expansion. Categorical variables included age, race/ethnicity, Elixhauser comorbidity index score, insurance payer, median residential income (eTable 4 in the Supplement), history of obesity (eTable 5 in the Supplement), history of diabetes, number of previous mental health diagnoses, history of smoking, history of irradiation, extended length of stay (>75th percentile), and presence of inpatient complications (eTable 6 in the Supplement). The data source provided race/ethnicity and we further categorized this variable into White, African American, Hispanic, and other racial/ethnic minority patients (Asian, Native American, and other racial/ethnic minority groups). Total charges were provided by the data source and used to estimate total inpatient costs using cost to charge ratios. Total inpatient costs represented fees incurred by the hospital from an inpatient stay, such as professional wages, operating room, utilities, and supplies.^27^ We included cost of care because changes in care costs can potentially affect the availability of services. In addition, we added interaction terms to examine whether expansion had unequal associations with different racial/ethnic groups. Assessing for interaction is critical in instances where the influence of an independent variable on an outcome can depend on a second independent variable,^28,29^ such as the association of expansion and reconstruction can vary across different patient demographic factors.

### Statistical Analysis

This study used difference-in-differences (DID) analysis, which is a pre-post study design frequently used in public policy research to evaluate how state-level policies are associated with the economy, health care, and society.^30,31,32,33^ DID analyses are employed in natural experiments when randomization is unfeasible and require a control population that is not exposed to the policy to account for background changes that occur over time.^34^ Medicaid expansion provides the ideal conditions to conduct a natural experiment that compares outcomes between expansion (intervention) and nonexpansion (control) states for a variety of health conditions.^20,35,36^ In order to reliably perform a DID analysis, the parallel trends assumption must be satisfied. This assumption requires preintervention trends to be similar in both the expansion and nonexpansion study populations and ensures the nonexpansion population can serve as an appropriate comparison group for this analysis (eAppendix in the Supplement).^37^ Fulfillment of the parallel trends assumption controlled for time-fixed effects among groups so that differences in outcomes between the study populations in the postexpansion period can be primarily associated with the legislative policy.

We used a DID logistic regression model to evaluate the association between Medicaid expansion and the use of autologous breast reconstruction. Data were analyzed from June 1, 2020, through February 28, 2021. The DID model was adjusted for covariates and included parameters for time period (ie, before and after expansion) and interaction term (DID estimate). We conducted a sensitivity analysis using a washout period of 12 months (January 2014 to December 2014). Statistical significance was set at *P* < .05, with analysis of variance used for continuous variables and χ^2^ test used for categorical variables. Data were analyzed using SAS statistical software version 9.4 (SAS Institute) with RStudio version 1.2.5033 and R software version 3.6.2 (R Project for Statistical Computing).

## Results

### Rates of Mastectomy vs Reconstruction Following Medicaid Expansion

In an overall sample of 45 850 patients who underwent mastectomy and 9215 patients who underwent autologous breast reconstruction, 36 777 patients (67%) were White and 32 205 (59%) had private insurance carriers (Table 1). Parallel trends test indicated no significant difference for autologous breast reconstruction during the preexpansion period between states that did and did not undergo expansion (eFigure 2 and eTable 7 in the Supplement). Table 1 shows the unadjusted differences between patients who underwent mastectomy or postmastectomy autologous reconstruction between the pre– and post–Medicaid expansion periods. From the preexpansion to postexpansion time period, the proportion of patients who underwent postmastectomy reconstruction increased from 18.1% (4951 of 27 290) to 23.0% (4264 of 18 560). Demographic characteristics of patients who underwent mastectomy with and without reconstruction were significantly different with regard to mean (SD) age (with reconstruction: 58 [13] years vs without: 57 [13] years; *P* < .001), racial/ethnic distribution (White [with: 68% vs without: 66%], African American [with: 13% vs without: 14%], Hispanic [with: 9% vs without: 10%], other racial/ethnic minority groups [with: 10% vs without: 10%]; *P* < .001), primary insurance payer (Medicare [with: 31% vs without: 29%], Medicaid [with: 9% vs without: 11%], private [with: 55% vs without: 56%], self-pay [with: 2% vs without: 1%], other [with: 2% vs without: 2%]; *P* < .001), and median residential income (quartile 4 [with: 37% vs without: 36%], quartile 3 [with: 24% vs without: 23%], quartile 2 [with: 21% vs without: 21%], quartile 1 [with: 18% vs without: 20%]; *P* < .001) between the preexpansion and postexpansion periods (Table 1). In our reconstructive cohort, the median residential income of patients was significantly different before and after expansion (quartile 4 [before expansion: 44% vs after: 41%], quartile 3 [before: 24% vs after: 23%], quartile 2 [before: 18% vs after: 20%], quartile 1 [before: 14% vs after: 16%]; *P* = .004). There were no significant differences with regards to reconstructive patients’ age, primary insurance type, and racial/ethnic distribution between the preexpansion and postexpansion period.

**Table 1.** Characteristics of Patients Who Underwent Mastectomy and Autologous Breast Reconstruction

Variable	Mastectomy			Postmastectomy autologous breast reconstruction		
	Preexpansion	Postexpansion	*P* value	Preexpansion	Postexpansion	*P* value
No.	27 290	18 560	NA	4951	4264	NA
Continuous variables						
Age, mean (SD), y	58 (13)	57 (13)	<.001^a^	53 (10)	53 (10)	.19
Categorical variables						
Race/ethnicity, No. (%)						
White	18 593 (68)	12 188 (66)	<.001^a^	3191 (64)	2805 (66)	.28
African American	3563 (13)	2613 (14)		736 (15)	624 (15)	
Hispanic	2399 (9)	1893 (10)		482 (10)	419 (10)	
Other^b^	2735 (10)	1866 (10)		542 (11)	416 (10)	
Median resident income, quartile, No. (%)^c^						
4	9995 (37)	6700 (36)	<.001^a^	2170 (44)	1769 (41)	.004^a^
3	6575 (24)	4227 (23)		1187 (24)	971 (23)	
2	5829 (21)	3965 (21)		912 (18)	854 (20)	
1	4891 (18)	3668 (20)		682 (14)	670 (16)	
Insurance payer^d^						
Medicare	8539 (31)	5372 (29)	<.001^a^	669 (14)	606 (14)	.27
Medicaid	2531 (9)	2120 (11)		439 (9)	406 (10)	
Private	15 126 (55)	10 371 (56)		3628 (73)	3080 (72)	
Self-pay	455 (2)	262 (1)		67 (1)	47 (1)	
Other	639 (2)	435 (2)		118 (2)	125 (3)	

Abbreviation: NA, not applicable.

^a^ Significant difference between the preexpansion period compared to the postexpansion period at 95% confidence interval; analysis of variance for continuous variables and χ^2^ test for categorical variables.

^b^ Other racial/ethnic minority groups included Asian, Native American, and other racial/ethnic minority groups.

^c^ US dollar ranges for quartiles vary by year in NIS (eTable 4 in the Supplement).

^d^ Private insurance category includes Blue Cross, commercial carriers, private health maintenance organizations, and preferred provider organizations. Other insurance category includes worker’s compensation, Civilian Health and Medical Program of the Uniformed Services, Civilian Health and Medical Program of the Department of Veterans Affairs, Title V of the McKinney-Vento Homeless Assistance Act, and other government programs.

### Cohort Characteristics of Patients Undergoing Autologous Reconstruction

Of 9215 patients with breast cancer who underwent autologous breast reconstruction, the 5996 patients (65.1%) were White and 6708 (72.8%) had private insurance (Table 2). For patients living in states that did not undergo Medicaid expansion, we observed significant differences in median residential income after 2014. There were no statistically significant differences in age, racial/ethnic distribution, primary payer, or residential income for patients living in expansion states before or after Medicaid expansion. In both expansion and nonexpansion states, there was a significant increase in the number of patients with obesity (expansion: 10% vs 15%; *P* < .001; χ^2^_1_ = 11.78; nonexpansion: 9% vs 12%; *P* = .004; χ^2^_1_ = 8.20), history of smoking (expansion: 16% vs 22%; *P* < .001; χ^2^_1_ = 15.56; nonexpansion: 19% vs 22%; *P* = .003; χ^2^_1_ = 8.50), and prior irradiation (expansion: 15% vs 18%; *P* = .008; χ^2^_1_ = 6.87; nonexpansion: 6% vs 8%, *P* = .004; χ^2^_1_ = 7.83) after 2014. The incidence of extended length of stay (24% vs 20%; *P* < .001; χ^2^_1_ = 16.13) was significantly lower in expansion states after expansion, whereas there were no differences in states that did not undergo expansion. Cost of care was significantly higher after expansion ($22 346 vs $23 211; *P* = .02) in states that did undergo expansion.

**Table 2.** Characteristics of Patients Who Underwent Postmastectomy Autologous Breast Reconstruction

Variable	States that did not undergo expansion			States that underwent expansion		
	Patients, No. (%)		*P* value	Patients, No. (%)		*P* value
	Preexpansion	Postexpansion		Preexpansion	Postexpansion	
No.	1642	1517		3,309	2,747	
Age, y						
18-29	13 (1)	12 (1)	.09	16 (1)	14 (1)	.63
30-39	117 (7)	128 (8)		243 (7)	180 (7)	
40-49	499 (30)	438 (29)		998 (30)	828 (30)	
50-59	585 (36)	495 (33)		1232 (37)	1000 (36)	
60-65	206 (13)	192 (13)		392 (12)	356 (13)	
>65	222 (14)	252 (17)		428 (13)	369 (13)	
Race/ethnicity						
White	1103 (67)	992 (65)	.47	2088 (63)	1813 (66)	.09
African American	278 (17)	254 (17)		458 (14)	370 (13)	
Hispanic	213 (13)	216 (14)		269 (8)	203 (7)	
Other^a^	48 (3)	55 (4)		494 (15)	361 (13)	
Diabetes						
Yes	131 (8)	117 (8)	.78	237 (7)	200 (7)	.86
No	1511 (92)	(92)		3072 (93)	2547 (93)	
Diabetes with chronic complications						
Yes	10 (1)	5 (0)	.25	12 (0)	8 (0)	.63
No	1632 (99)	1512 (100)		3297 (100)	2739 (100)	
Obesity						
Yes	171 (10)	220 (15)	<.001^b^	313 (9)	323 (12)	.004^b^
No	1471 (90)	1297 (85)		2996 (91)	2424 (88)	
History of smoking						
Yes	263 (16)	327 (22)	<.001^b^	632 (19)	609 (22)	.003^b^
No	1379 (84)	1190 (78)		2677 (81)	2138 (78)	
Prior irradiation						
Yes	241 (15)	276 (18)	.008^b^	203 (6)	220 (8)	.004^b^
No	1401 (85)	1241 (82)		3106 (94)	2527 (92)	
Elixhauser Comorbidity Index score						
0-4	1618 (99)	1476 (97)	.01^b^	3252 (98)	2702 (98)	.80
≥5	24 (1)	41 (3)		57 (2)	45 (2)	
Mental health diagnosis						
None	1359 (83)	1180 (78)	<.001^b^	2700 (82)	2217 (81)	.38
>1	283 (17)	337 (22)		609 (18)	530 (19)	
Median residential income, quartile^c^						
4	332 (20)	260 (17)	<.001^b^	1838 (56)	1509 (55)	.44
3	485 (30)	402 (26)		702 (21)	569 (21)	
2	486 (30)	461 (30)		426 (13)	393 (14)	
1	339 (21)	394 (26)		343 (10)	276 (10)	
Insurance payer^d^						
Medicare	257 (16)	261 (17)	.09	442 (13)	345 (13)	.21
Medicaid	98 (6)	114 (8)		341 (10)	292 (11)	
Private	1186 (72)	1062 (70)		2442 (74)	2018 (73)	
Self-pay	32 (2)	17 (1)		35 (1)	30 (1)	
Other	69 (4)	63 (4)		49 (2)	62 (2)	
Inpatient complications						
None	1525 (93)	1404 (93)	.73	3083 (93)	2687 (94)	.11
Any	117 (7)	113 (7)		226 (7)	160 (6)	
Extended length of stay						
Yes	509 (31)	425 (28)	.07	795 (24)	541 (20)	<.001^b^
No	1133 (69)	1092 (72)		2514 (76)	2206 (80)	
Continuous variables, mean (SD)						
Inpatient costs, $	23 312 (12 625)	24 132 (13 515)	.17	22 346 (13 251)	23 211 (13 579)	.02^b^

^a^ Other racial/ethnic minority groups included Asian, Native American, and other racial/ethnic minority groups.

^b^ Significant difference between the occurrence of postmastectomy breast reconstruction in the preexpansion period compared to the postexpansion period at 95% confidence interval; analysis of variance for continuous variables and χ^2^ test for categorical variables.

^c^ US dollar ranges for quartiles vary by year in NIS (eTable 4 in the Supplement).

^d^ Private insurance category includes Blue Cross, commercial carriers, private health maintenance organizations, and preferred provider organizations. Other insurance category includes worker’s compensation, Civilian Health and Medical Program of the Uniformed Services, Civilian Health and Medical Program of the Department of Veterans Affairs, Title V of the McKinney-Vento Homeless Assistance Act, and other government programs.

### Trends in Postmastectomy Breast Reconstruction

In this DID analysis, we evaluated the association between Medicaid expansion and autologous breast reconstruction through comparing the influence of expansion (ie, the intervention) on an outcome by comparing the average change over time of patients in the treatment group (ie, patients living in expansion states after expansion) compared to the control group (ie, patients living in nonexpansion states). Table 3 shows the results across various demographic variables. Compared with 2012, the odds of autologous reconstruction were higher in subsequent years (2013: odds ratio [OR], 1.22; 95% CI, 1.15-1.30; *P* < .001; 2014: OR, 1.55; 95% CI; 1.41-1.71; *P* < .001; 2015: OR, 1.64; 95% CI, 1.48-1.80; *P* < .001). The odds of reconstruction were lower for patients with comorbidities, such as those with diabetes (OR, 0.68; 95% CI, 0.61-0.75; *P* < .001) and diabetes with chronic complications (OR, 0.57; 95% CI; 0.39-0.83; *P* = .003). African American (OR, 1.43; 95% CI, 1.33-1.55; *P* < .001) and Hispanic (OR, 1.44; 95% CI, 1.31-1.60; *P* < .001) patients were associated with higher odds of reconstruction compared with White patients over our study period. With the highest residential income as the reference, the odds of reconstruction were significantly lower in all other income quartiles. Compared with patients with private insurance, those with Medicare (OR, 0.61; 95% CI, 0.56-0.68; *P* < .001) and Medicaid (OR, 0.62; 95% CI, 0.58-0.68; *P* < .001) had lower odds of receiving reconstructive surgical procedures. These findings suggest positive temporal changes across all states; however, patient demographic characteristics, primary payer, and income remain influential factors associated with the receipt of surgery.

**Table 3.** Results of the Multivariable Logistic Regression Analysis Examining the Probability of Autologous Breast Reconstruction

Variable	OR (95% CI)	*P* value
Age, y		
18-29	1 [Reference]	NA
30-39	1.59 (1.16-2.17)	.003^a^
40-49	1.94 (1.44 − 2.60)	<.001^a^
50-59	2.01 (1.50-2.70)	<.001^a^
60-65	1.54 (1.15-2.06)	.005^a^
65+	0.81 (0.59-1.11)	.18
Race/ethnicity		
White	1 [Reference]	NA
African American	1.43 (1.33-1.55)	<.001^a^
Hispanic	1.44 (1.31-1.60)	<.001^a^
Other^b^	1.08 (0.98-1.19)	.15
Year		
2012	1 [Reference]	NA
2013	1.22 (1.15-1.30)	<.001^a^
2014	1.55 (1.41-1.71)	<.001^a^
2015	1.64 (1.48-1.80)	<.001^a^
State		
Florida^c^	1 [Reference]	NA
North Carolina^c^	1.53 (1.38-1.68)	<.001^a^
Wisconsin^c^	1.28 (1.14-1.44)	<.001^a^
New Jersey^d^	1.04 (0.93-1.14)	.49
New York^d^	1.53 (1.41-1.65)	<.001^a^
Washington^d^	0.95 (0.85-1.07)	.43
Diabetes		
Yes	0.68 (0.61-0.75)	<.001^a^
No	1 [Reference]	NA
Diabetes with chronic complications		
Yes	0.57 (0.39-0.83)	.003^a^
No	1 [Reference]	NA
Obesity		
Yes	1.29 (1.20-1.40)	<.001^a^
No	1 [Reference]	NA
History of smoking		
Yes	1.04 (0.98-1.10)	.19
No	1 [Reference]	NA
Prior irradiation		
Yes	4.26 (3.87-4.70)	<.001^a^
No	1 [Reference]	NA
Elixhauser Comorbidity Index		
0-4	1 [Reference]	NA
>5	0.79 (0.67-0.95)	.01^a^
Mental health diagnosis		
None	1 [Reference]	NA
>1	1.18 (1.11-1.24)	<.001^a^
Median residential income, quartile^e^		
4	1 [Reference]	NA
3	0.86 (0.81-0.91)	<.001^a^
2	0.80 (0.73-0.86)	<.001^a^
1	0.69 (0.64-0.75)	<.001^a^
Insurance payer^f^		
Private	1 [Reference]	
Medicare	0.61 (0.56-0.68)	<.001^a^
Medicaid	0.62 (0.58-0.68)	<.001^a^
Self-pay	0.59 (0.47-0.73)	<.001^a^
Other	0.93 (0.80-1.09)	.35
Interaction term (expansion)		
White	0.94 (0.84-1.06)	.26
African American	0.72 (0.61-0.87)	<.001^a^
Hispanic	0.60 (0.50-0.74)	<.001^a^
Other^b^	0.80 (0.67-0.96)	.01^a^

Abbreviations: NA, not applicable; OR, odds ratio.

^a^ Significant difference at 95% confidence interval.

^b^ Other racial/ethnic minority groups included Asian, Native American, and other racial ethnic minority groups.

^c^ State that did not undergo expansion.

^d^ State that underwent expansion.

^e^ US dollar ranges for quartiles vary by year in NIS (eTable 4 in the Supplement).

^f^ Private insurance category includes Blue Cross, commercial carriers, private health maintenance organizations, and preferred provider organizations. Other insurance category includes worker’s compensation, Civilian Health and Medical Program of the Uniformed Services, Civilian Health and Medical Program of the Department of Veterans Affairs, Title V of the McKinney-Vento Homeless Assistance Act, and other government programs.

### Association Between Medicaid Expansion and Reconstruction by Racial/Ethnic Groups

The ACA’s Medicaid expansion exhibited unequal implications among racial/ethnic groups. Expansion was not significantly associated with changes to the odds of reconstruction for White patients (OR, 0.94; 95% CI, 0.84-1.06; *P* = .26), yet was associated with statistically significant decreases of 28% for African American patients (OR, 0.72; 95% CI, 0.61–0.87; *P* < .001), 40% for Hispanic patients (OR, 0.60; 95% CI, 0.50-0.74; *P* < .001), and 20% for patients from other racial/ethnic minority groups (OR, 0.80; 95% CI, 0.67-0.96; *P* = .01) (Table 3). Sensitivity analysis including a 12-month washout period demonstrated similar results (eTable 8 in the Supplement). These findings suggest that the odds of reconstruction for non-White patients were lowered in Medicaid-expansion states, potentially demonstrating existing disparities in access for autologous breast reconstruction.

## Discussion

Through a difference-in-differences analysis of the State Inpatient Database, we examined the association between Medicaid expansion and the use of autologous breast reconstruction in patients with breast cancer. The use of autologous breast reconstruction increased annually throughout our study period, with patients possessing higher income, private insurance, or having African American or Hispanic ethnicity demonstrating higher odds of undergoing autologous reconstructive surgery overall. However, comparison of expansion and nonexpansion states were associated with unequal outcomes on the use of reconstruction among racial/ethnic groups. There was no significant change in the rates of reconstruction for White patients, whereas patients of color were less likely to undergo reconstructive surgery. This study adds to the body of evidence of downstream implications of national health care policies and their ability to further existing racial/ethnic disparities in the use of autologous breast reconstruction.

The ACA Medicaid expansion provision increased insurance coverage, yet our study found pervasive disparities in the use of autologous breast reconstruction. These findings suggest that factors outside of hospital costs and care outcomes influence the decision regarding breast reconstruction. Patients could be hindered by direct expenses, such as high premiums and out-of-pocket payments, as well as indirect expenses, such as longer time away from work, hospitalization, and operative time. The time-intensive surgery and recovery process could substantially deter disadvantaged patients with limited resources.

Race/ethnicity and household income have been previously identified as significant negative predictors of receipt of autologous breast reconstruction.^7,38,39,40^ Despite efforts to reduce racial/ethnic and socioeconomic disparities in the receipt of postmastectomy reconstruction, this analysis suggests few benefits of Medicaid expansion on the use of autologous breast reconstruction. Our results are in agreement with findings from prior studies indicating that less than 20% of postmastectomy patients undergo autologous breast reconstruction despite well-documented long-term benefits.^10,41^ Factors that may contribute to these observations include the lack of physician knowledge about psychosocial benefits, insufficient access to a specialty center performing autologous breast reconstruction, and deficiencies in the clinician-patient relationship. Furthermore, individuals who are uninsured and underinsured are more likely to delay seeking care, often presenting with late pathologies or more severe disease, which may affect a clinician’s discussion of breast reconstruction.^42,43^ There may be a mismatch between patients and health care clinicians’ perceptions of breast reconstruction outcomes.^44^ In 2007, fewer than one-fourth of general surgeons referred more than 75% of their patients for reconstruction consultation prior to undergoing mastectomy.^45^ From the patient perspective, only a few reported having enough knowledge to make informed treatment decisions after communicating interest to their surgeon.^44,46^ This suggests that awareness of breast reconstruction has increased, yet detailed knowledge regarding the procedure and health outcomes remain low.^46^ Furthermore, perceived limited availability of plastic surgeons by other health care professionals has been associated with decreased odds of reconstruction referral.^40,45^ In particular, autologous breast reconstruction requires specialized surgeons trained in microsurgery and care facilities with microsurgical equipment. Breast reconstruction rates are significantly higher at well-equipped academic centers,^47^ but patients must travel farther for autologous reconstruction at these facilities compared with more readily available community hospitals.^48^ Moreover, surgical decision-making can be biased by surgeon availability. For example, communities with more plastic surgeons who prefer implant-based reconstructions can result in fewer patients receiving autologous reconstruction. The variation in clinicians and services can deter patients and further promote geographic disparities. Potential solutions include improving the availability of plastic surgeons and incorporation of teleconsultation, negating the financial and logistical difficulties of travel.^40^

Patients of color have historically experienced disproportionate barriers to access and reduced quality of care, contributing to disparities in health outcomes.^49,50^ Despite health policy programs and policies targeted at improving coverage, obtaining insurance is not sufficient for ensuring access and high quality of care owing to multitudes of challenges, such as insurance enrollment, consistent care with a primary care physician, and low health literacy.^51,52,53^ Changes in other aspects of care delivery and expanding coverage are required to decrease disparities in care and health outcomes. Moreover, evaluating changes introduced by public health programs and policies is a first and essential step in ensuring fulfillment of intended purposes and identifying persistent barriers to further health policy reform.

### Limitations

This study had several limitations inherent to the use of administrative claims data. First, database analysis is dependent on accuracy of the data captured. This database does not follow patients longitudinally and will not capture complications occurring outside of the initial hospital admission or 30-day readmission rates. Moreover, this database does not capture information such as patient preferences and clinician attitudes toward autologous breast reconstruction, or the information they are given about these procedures. Second, this study specifically analyzed autologous breast reconstruction and is not generalizable to other forms of breast reconstruction such as implant-based reconstructions. Further investigation is needed to evaluate the relationship between Medicaid expansion and other methods of breast reconstruction. Third, total inpatient charges were utilized to estimate the cost of care. This price will differ from the total payments collected from the primary payer (ie, insurance company and/or patient). However, prior studies using complete financial data from a subset of the Health Care Utilization Project’s National Inpatient Sample hospitals concluded that the cost-to-charge ratios are highly correlated with payment-to-charge estimates.^54^ Fourth, DID analyses are inherent to selection bias, which can limit internal validity. We reduced this by adjusting for confounding variables in our regression model. More importantly, we evaluated for parallel trends to ensure model validity. Though we cannot ascertain how closely the model resembles real life events, DID analysis remains a useful analytical tool to evaluate changes in health policy. Lastly, we limited our time frame through the third quartile of 2015 because of the coding transition from *ICD-9* to *ICD-10*, which led to a 5-fold increase in available codes and could lead to errors in coding or increased variation in coding practices.^55,56^

## Conclusions

In this cross-sectional study, our analysis suggests that Medicaid expansion was not associated with increasing rates of autologous breast reconstruction. Furthermore, Medicaid expansion was associated with decreasing the odds of autologous reconstruction for patients of color. Autologous breast reconstruction is a specialized area of care with demonstrated patient benefits. Further exploration into the factors hindering access and development of effective strategies to combat them for historically marginalized groups is essential.

## Notes

Supplement. **eAppendix.** Difference-in-differences Model **eFigure 1. **Schematic Flowchart of Patients Included in Multivariable Regression Analysis **eFigure 2. **Tests of Parallel Trends for Autologous Breast Reconstruction **eTable 1. **ICD-9 Diagnostic Codes **eTable 2. **ICD-9 Procedure Codes **eTable 3. **Characteristics of Total Patients Included in Multivariable Regression Analysis and Excluded Patients Owing to Missing Values **eTable 4. **Quartile Ranges for Median Household Income by Year in the State Inpatient Database **eTable 5. **Patient Comorbidities by ICD-9 Diagnostic Codes **eTable 6. **Complications by ICD-9 Diagnostic Codes **eTable 7. **Comparison of Patient Demographic Characteristics in the Pre-expansion Period **eTable 8. **Results of the Multivariable Logistic Regression
