# Errors in Tables

**DOI:** 10.1001/jamanetworkopen.2022.11388

**Published:** 2022-04-18

In the Original Investigation titled “Factors Associated With State-Specific Medicaid Expansion and Receipt of Autologous Breast Reconstruction Among Patients Undergoing Mastectomy,”^1^ published August 3, 2021, Table 2 of the main article and eTable 7 of the Supplement contained cells with fewer than 10 participants. These cells should have been redacted to comply with data use guidelines regarding patient privacy. This article has been corrected.^1^
