# Supplementary material for: Factors Associated With State-Specific Medicaid Expansion and Receipt of Autologous Breast Reconstruction Among Patients Undergoing Mastectomy
Source: JAMA Netw Open. 2021 Aug 3;4(8):e2119141. doi: 10.1001/jamanetworkopen.2021.19141 (PMC8335577; doi:10.1001/jamanetworkopen.2021.19141)
Supplement: Supplement. — eAppendix. Difference-in-differences Model eFigure 1. Schematic Flowchart of Patients Included in Multivariable Regression Analysis eFigure 2. Tests of Parallel Trends for Autologous Breast Reconstruction eTable 1. ICD-9 Diagnostic Codes eTable 2. ICD-9 Procedure Codes eTable 3. Characteristics of Total Patients Included in Multivariable Regression Analysis and Excluded Patients Owing to Missing Values eTable 4. Quartile Ranges for Median Household Income by Year in the State Inpatient Database eTable 5. Patient Comorbidities by ICD-9 Diagnostic Codes eTable 6. Complications by ICD-9 Diagnostic Codes eTable 7. Comparison of Patient Demographic Characteristics in the Pre-expansion Period eTable 8. Results of the Multivariable Logistic Regression [file jamanetwopen-e2119141-s001.pdf]

Supplemental Online Content

Huynh KA, Jayaram M, Wang C, et al. Factors associated with state-specific Medicaid expansion and receipt of autologous breast reconstruction among patients undergoing mastectomy. *JAMA Netw Open.* 2021;4(8):e2119141.
doi:10.1001/jamanetworkopen.2021.19141

eAppendix. Difference-in-differences Model

eFigure 1. Schematic Flowchart of Patients Included in Multivariable Regression Analysis

eFigure 2. Tests of Parallel Trends for Autologous Breast Reconstruction

eTable 1. ICD-9 Diagnostic Codes

eTable 2. ICD-9 Procedure Codes

eTable 3. Characteristics of Total Patients Included in Multivariable Regression Analysis and Excluded Patients Owing to Missing Values

eTable 4. Quartile Ranges for Median Household Income by Year in the State Inpatient Database

eTable 5. Patient Comorbidities by ICD-9 Diagnostic Codes

eTable 6. Complications by ICD-9 Diagnostic Codes

eTable 7. Comparison of Patient Demographic Characteristics in the Pre-expansion Period

eTable 8. Results of the Multivariable Logistic Regression

This supplemental material has been provided by the authors to give readers additional information about their work.

© 2021 Huynh KA et al. *JAMA Network Open.*

eAppendix. Difference-in-differences Model

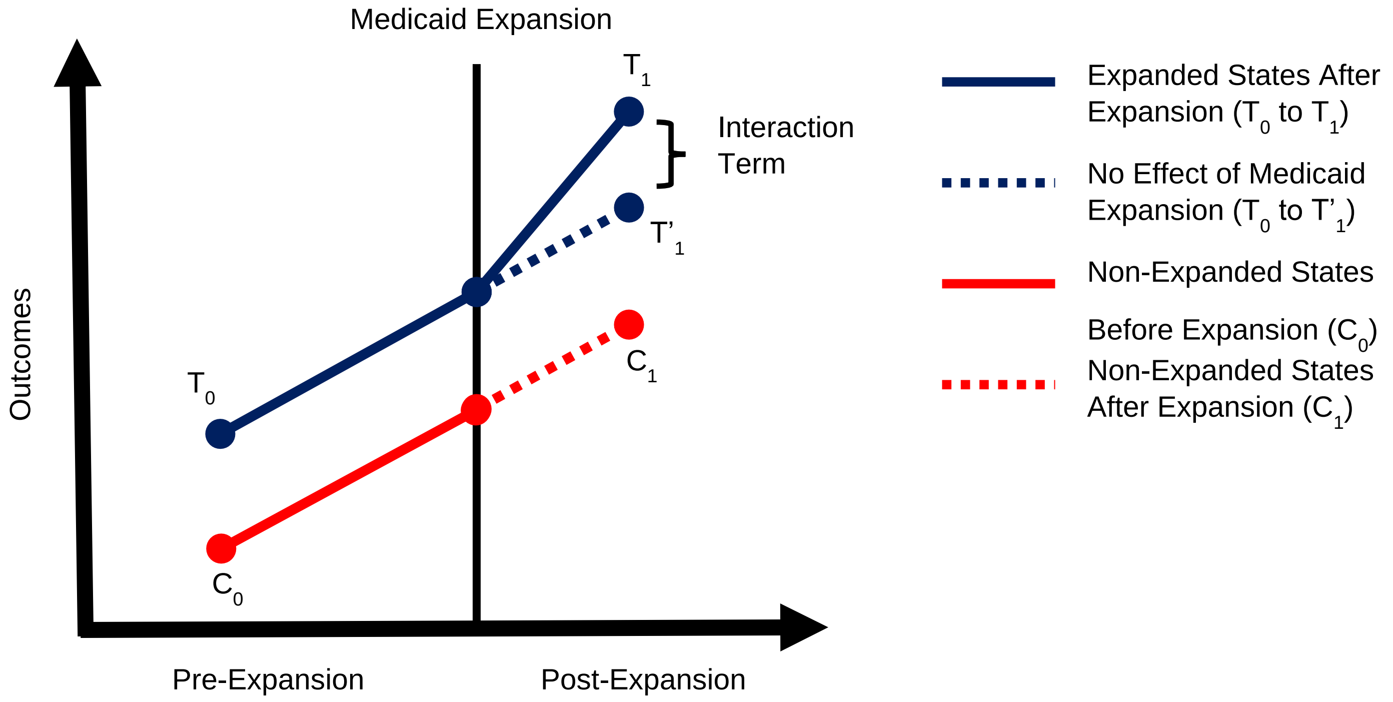

Difference-in-differences assumes the trends in outcomes (e.g. readmission cost) would be similar (slopes) for both expanded (T_0 to T'_1) and non-expanded states (C_0 to C_1) if there was no effect of Medicaid expansion. The interaction term estimates the effect of expansion by comparing post-intervention outcomes in expanded states with predicted outcomes if no expansion had occurred. Here, the interaction term is calculated for two groups in two distinct time periods. We can also calculate the combined interaction terms for "expansion and race" to investigate how Medicaid expansion influences outcomes for different races.

© 2021 Huynh KA et al. *JAMA Network Open.*

eFigure 1. Schematic Flowchart of Patients Included in Multivariable Regression Analysis

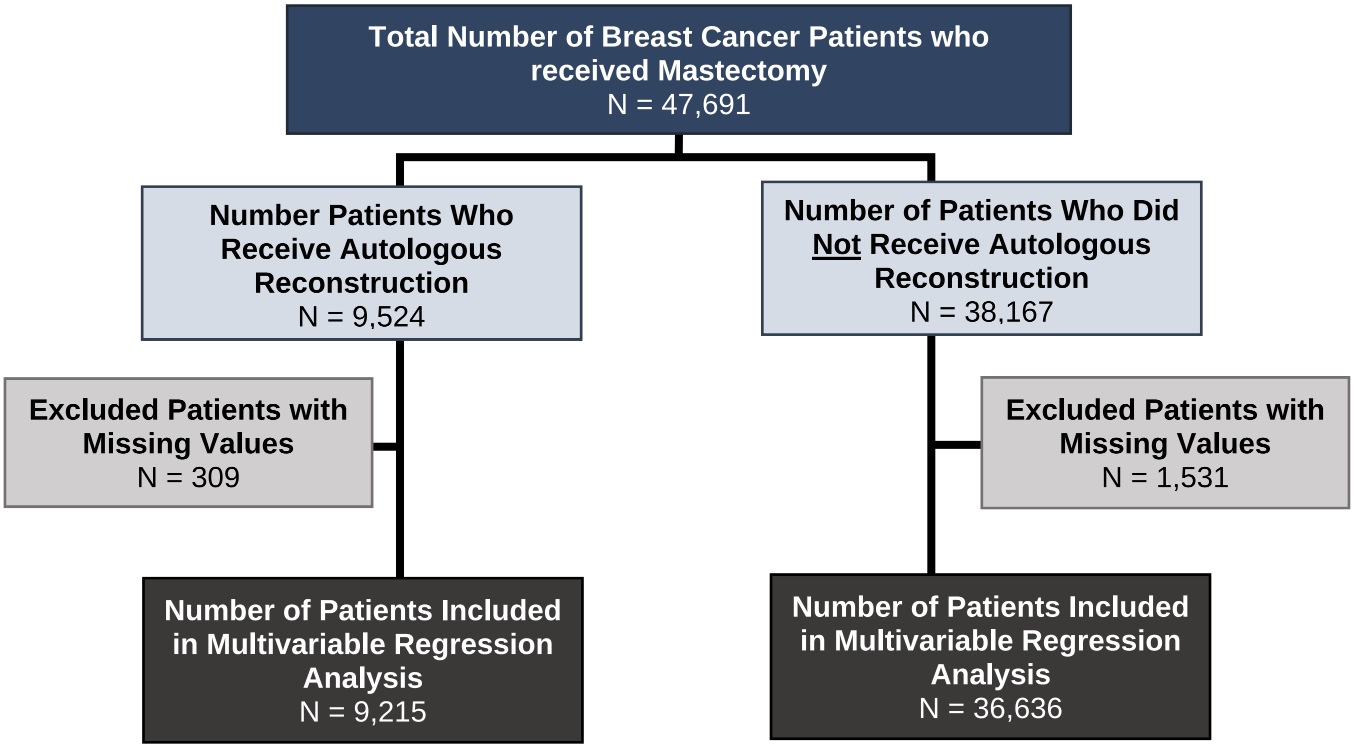

Source: The 2012-2015 Healthcare Cost and Utilization Project State Inpatient Databases from New York, Washington, New Jersey, Florida, North Carolina, and Wisconsin.

© 2021 Huynh KA et al. *JAMA Network Open.*

eFigure 2. Tests of Parallel Trends for Autologous Breast Reconstruction

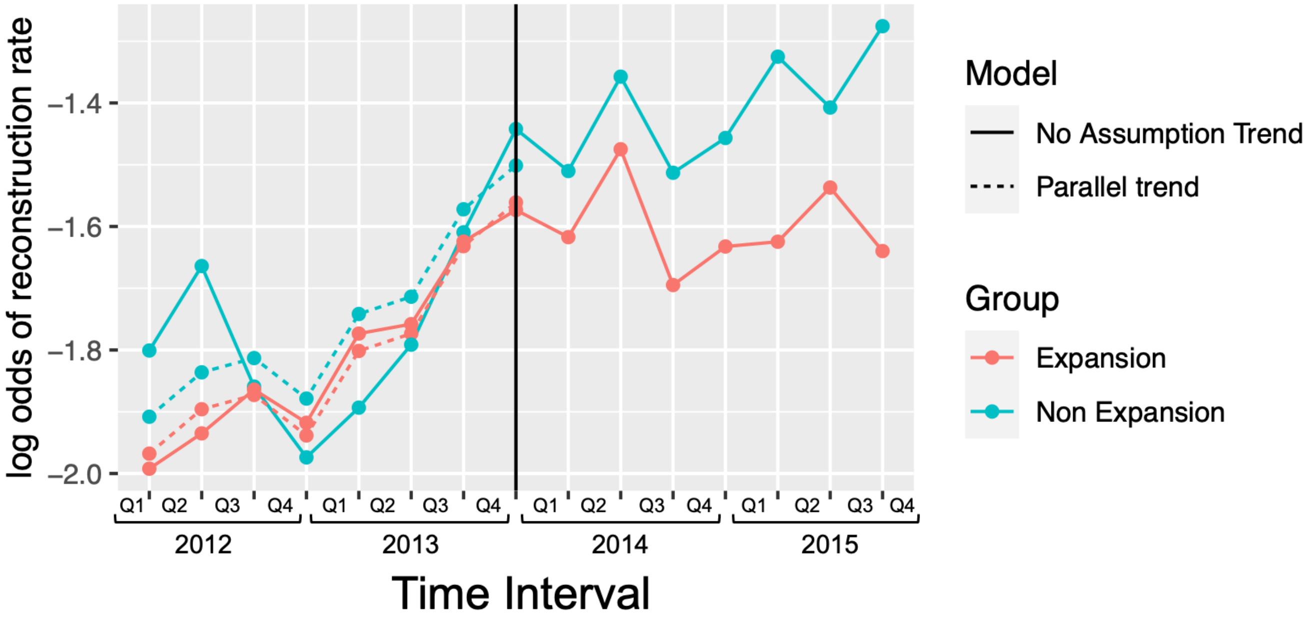

Parallel trends test demonstrates similar trends for autologous breast reconstruction between states that did and did not undergo expansion prior to 2014 (P-value = 0.43).

© 2021 Huynh KA et al. *JAMA Network Open.*

eTable 1. ICD-9 Diagnostic Codes

Description	ICD-9 CM
**Breast Cancer Diagnosis**	
Malignant neoplasm of female breast	174.0<br>174.1<br>174.2<br>174.3<br>174.4<br>174.5<br>174.6<br>174.8<br>174.9
Carcinoma in situ of breast	233.0
Neoplasm of uncertain behavior of other and unspecified sites and tissues: Breast	283.3
Neoplasms of unspecified nature: breast	239.3

© 2021 Huynh KA et al. *JAMA Network Open.*

eTable 2. ICD-9 Procedure Codes

Description	ICD-9 PCS
**Mastectomy**	
Other unilateral subcutaneous mammectomy	85.34
Other bilateral subcutaneous mammectomy	85.36
Mastectomy	85.4
Unilateral simple mastectomy	85.41
Bilateral simple mastectomy	85.42
Unilateral extended simple mastectomy	85.43
Bilateral extended simple mastectomy	85.44
Unilateral radical mastectomy	85.45
Bilateral radical mastectomy	85.46
Unilateral extended radical mastectomy	85.47
Bilateral extended radical mastectomy	85.48
**Breast Reconstruction Procedures**	
Total reconstruction, unspecified	85.70
Latissimus Dorsi Myocutaneous Flap	85.71
TRAM, pedicle	85.72
TRAM, free	85.73
DIEP, free	85.74
SIEA, free	85.75
GAP, free	85.76
Pedicle graft to breast	85.84
Muscle flap graft to breast	85.85

© 2021 Huynh KA et al. *JAMA Network Open.*

eTable 3. Characteristics of Total Patients Included in Multivariable Regression Analysis and Excluded Patients Owing to Missing Values

	Total Included Patients (N = 45,851)		Patients with Missing Values (N = 1,840)		P-value
**Continuous Variables**	**Mean**	**(SD)**	**Mean**	**(SD)**	
Age, years	58	(13)	56	(13)	<0.001*
**Categorical Variables**	**N**	**(%)**	**N**	**(%)**	
Year					<0.001*
2012	14,837	(32)	537	(29)	
2013	12,453	(27)	547	(30)	
2014	10,872	(24)	530	(29)	
2015	7,689	(17)	226	(12)	
Race					<0.001*
White	30,781	(67)	508	(28)	
Black	6,176	(13)	397	(22)	
Hispanic	4,292	(10)	244	(13)	
Other Minorities[a]	4,602	(10)	275	(15)	
Missing	0	(0)	416	(25)	
Residential Income[b]					<0.001*
Quartile 1	8,560	(19)	78	(4)	
Quartile 2	9,794	(21)	136	(7)	
Quartile 3	10,802	(24)	152	(8)	
Quartile 4	16,695	(36)	160	(9)	
Missing	0	(0)	1,314	(71)	
Insurance[c]					<0.001*
Medicare	13,911	(30)	433	(24)	
Medicaid	4,651	(10)	640	(35)	
Private	25,497	(56)	654	(36)	
Self-pay	717	(2)	58	(3)	
Other	1,075	(2)	32	(2)	
Missing	0	(0)	23	(1)	

**Indicates a significant difference between total patients included in multivariable regression analysis and excluded patients owing to missing values at 95% confidence level; Welch's Two Sample t-test for continuous variables and Chi-squared test for categorical variables.*
[a] Other Minorities includes Asians, Native Americans, and Other Minority groups
[b] U.S. dollar ranges for quartiles vary by year in NIS (eTable 4).
[c] Private insurance category includes Blue Cross, commercial carriers, private Health Maintenance Organizations, and Preferred Provider Organizations. Other insurance category includes worker's compensation, CHAMPUS, CHAMPVA, Title V of the McKinney-Vento Homeless Assistance Act, and other government programs.

© 2021 Huynh KA et al. *JAMA Network Open.*

eTable 4. Quartile Ranges for Median Household Income by Year in the State Inpatient Database

Year	Quartile 1 ($)	Quartile 2 ($)	Quartile 3 ($)	Quartile 4 ($)
2012	1 - 38,999	39,000 - 47,999	48,000 - 62,999	63,000+
2013	1 - 37,999	38,000 - 47,999	48,000 - 63,999	64,000+
2014	1 - 39,999	40,000 - 50,999	51,000 - 65,999	66,000+
2015	1 - 41,999	42,000 - 51,999	52,000 - 67,999	68,000+

© 2021 Huynh KA et al. *JAMA Network Open.*

eTable 5. Patient Comorbidities by ICD-9 Diagnostic Codes

Description	ICD-9 CM
Obesity	278.0<br>278.00<br>278.01<br>278.02<br>278.03
Diabetes	25000<br>25001<br>25002<br>25003<br>25010<br>25011<br>25012<br>25013<br>25020<br>25021<br>25022<br>25023<br>25030<br>25031<br>25032<br>25033<br>25040<br>25041<br>25042<br>25043<br>25050<br>25051<br>25052<br>25053<br>25060<br>25061<br>25062<br>25063<br>25070<br>25071<br>25072<br>25073<br>25080<br>25081<br>25082<br>25083<br>25090<br>25091<br>25092<br>25093
History of smoking	305.1<br>V15.82
Prior irradiation	V15.3

© 2021 Huynh KA et al. *JAMA Network Open.*

eTable 6. Complications by ICD-9 Diagnostic Codes

Surgical	ICD-9 CM
Wound dehiscence	998.3<br>998.30<br>998.31<br>998.32
Wound infection	998.5<br>998.51<br>998.59
Hemorrhage/Hematoma/Seroma	998.1<br>998.11<br>998.12<br>998.13
Micro anastomotic complications	39.41<br>39.49<br>39.30<br>39.31<br>39.32<br>39.56<br>39.57<br>39.58<br>39.59
Post-operative shock	998.09<br>998.00<br>998.01<br>998.02
Blood transfusion	99.00<br>99.03<br>99.04<br>v58.2
**Medical**	**ICD-9 CM**
Respiratory failure/Pneumonia	518.81<br>518.4<br>518.51<br>518.52<br>518.84<br>480<br>481<br>482<br>483<br>484<br>485<br>486<br>480.8<br>482.8<br>507.0<br>507.1<br>507.8
Stroke	997.02

© 2021 Huynh KA et al. *JAMA Network Open.*

eTable 6. Complications by ICD-9 Diagnostic Codes (continue)

Medical	ICD-9 CM
Myocardial Infarction	410<br>410.0<br>410.00<br>410.01<br>410.02<br>410.1<br>410.10<br>410.11<br>410.12<br>410.2<br>410.20<br>410.21<br>410.22<br>410.3<br>410.30<br>410.31<br>410.32<br>410.4<br>410.40<br>410.41<br>410.42<br>410.5<br>410.50<br>410.51<br>410.52<br>410.6<br>410.60<br>410.61<br>410.62<br>410.7<br>410.70<br>410.71<br>410.72<br>410.8<br>410.80<br>410.81<br>410.82<br>410.9<br>410.90<br>410.91<br>410.92
Urinary tract infection/Renal complications	997.5<br>584.5<br>584.6<br>584.7<br>584.8<br>584.9

© 2021 Huynh KA et al. *JAMA Network Open.*

eTable 6. Complications by ICD-9 Diagnostic Codes (continue)

Medical	ICD-9 CM
Deep venous thrombosis/ Pulmonary embolism	415.0<br>415.1<br>415.11<br>415.19<br>451.2<br>451.81<br>453.40<br>453.41<br>453.42<br>453.8<br>453.9

© 2021 Huynh KA et al. *JAMA Network Open.*

eTable 7. Comparison of Patient Demographic Characteristics in the Pre-expansion Period

	States that Did Not Undergo Expansion (N = 1,642)		States That Underwent Expansion (N = 3,309)		P-value
	N	(%)	N	(%)	
Age (years)					0.64
18-29	13	(1)	16	(1)	
30-39	117	(7)	243	(8)	
40-49	499	(30)	998	(29)	
50-59	585	(36)	1,232	(33)	
60-65	206	(13)	392	(13)	
65+	222	(14)	428	(17)	
Race					<0.001*
White	1,103	(67)	2,088	(65)	
Black	278	(17)	458	(17)	
Hispanic	213	(13)	269	(14)	
Other Minorities[a]	48	(3)	494	(4)	
Diabetes					0.30
Yes	131	(8)	237	(8)	
No	1,511	(92)	3,072	(92)	
Diabetes with Chronic Complications					0.22
Yes	10	(1)	12	(0)	
No	1,632	(99)	3,297	(100)	
Obesity					0.29
Yes	171	(10)	313	(15)	
No	1,471	(90)	2,996	(85)	
History of Smoking					0.008*
Yes	263	(16)	632	(22)	
No	1,379	(84)	2,677	(78)	
Prior Irradiation					<0.001*
Yes	241	(15)	203	(18)	
No	1,401	(85)	3,106	(82)	
Elixhauser Comorbidity Index					0.50
0-4	1,618	(99)	3,252	(97)	
$\geq$ 5	24	(1)	57	(3)	
Mental Health Diagnosis					0.31
None	1,359	(83)	2,700	(78)	
>1	283	(17)	609	(22)	
Median Residential Income[b]					0.29
Quartile 4	332	(20)	1,838	(17)	
Quartile 3	485	(30)	702	(26)	
Quartile 2	486	(30)	426	(30)	
Quartile 1	339	(21)	343	(26)	
Insurance Payer[c]					<0.001*
Medicare	257	(16)	442	(17)	
Medicaid	98	(6)	341	(8)	
Private	1,186	(72)	2,442	(70)	
Self-pay	32	(2)	35	(1)	
Other	69	(4)	49	(4)	

© 2021 Huynh KA et al. *JAMA Network Open.*

**eTable 7.** Comparison of Patient Demographic Characteristics in the Pre-expansion Period (continue)					
	**States that Did Not Undergo Expansion (N = 1,642)**		**States That Underwent Expansion (N = 3,309)**		**P-value**
	**N**	**(%)**	**N**	**(%)**	
Inpatient Complications					0.70
None	1,525	(93)	3,083	(93)	
Any	117	(7)	226	(7)	
Extended Length of Stay					<0.001*
Yes	509	(31)	795	(28)	
No	1,133	(69)	2,514	(72)	
**Continuous Variables**	**Mean**	**(SD)**	**Mean**	**(SD)**	
Average Inpatient Costs	$23,312	($12,625)	$22,346	($13,515)	<0.001*

** Indicates a significant difference between patients living in states that did and did not undergo expansion in the pre-expansion period at 95% confidence level; Welch's Two Sample t-test for continuous variables and Chi-squared test for categorical variables.*
[a] Other Minorities includes Asians, Native Americans, and Other Minority groups
[b] U.S. dollar ranges for quartiles vary by year in NIS (eTable 4).
[c] Private insurance category includes Blue Cross, commercial carriers, private Health Maintenance Organizations, and Preferred Provider Organizations. Other insurance category includes worker's compensation, CHAMPUS, CHAMPVA, Title V of the McKinney-Vento Homeless Assistance Act, and other government programs.

© 2021 Huynh KA et al. *JAMA Network Open.*

eTable 8. Results of the Multivariable Logistic Regression Analysis Examining the Probability of Autologous Breast Reconstruction

	OR	(95% Confidence Interval)			P-value
Age (years)					
18-29	1				
30-39	1.57	(1.10	-	2.23)	0.012[d]
40-49	1.95	(1.40	-	2.73)	<0.001[d]
50-59	2.03	(1.46	-	2.84)	<0.001[d]
60-65	1.55	(1.09	-	2.21)	0.014[d]
65+	0.81	(0.57	-	1.15)	0.26
Race					
White	1				
Black	1.42	(1.29	-	1.57)	<0.001[d]
Hispanic	1.42	(1.29	-	1.57)	<0.001[d]
Other Minorities[a]	1.07	(0.97	-	1.18)	0.21
Year					
2012	1				
2013	1.22	(1.15	-	1.30)	<0.001[d]
2015	1.73	(1.54	-	1.95)	<0.001[d]
State					
Florida	1				
North Carolina	1.60	(1.42	-	1.80)	<0.001[d]
Wisconsin	1.21	(1.05	-	1.39)	0.004[d]
New Jersey	1.08	(0.98	-	1.19)	0.16
New York	1.53	(1.42	-	1.66)	<0.001[d]
Washington	0.87	(0.76	-	1.00)	0.05
Diabetes					
Yes	0.67	(0.61	-	0.74)	<0.001[d]
No	1				
Diabetes with Chronic Complications					
Yes	0.70	(0.47	-	1.06)	0.10
No	1				
Obesity					
Yes	1.26	(1.14	-	1.39)	<0.001[d]
No	1				
History of Smoking					
Yes	1.01	(0.93	-	1.09)	0.78
No	1				
Prior Irradiation					
Yes	4.52	(4.02	-	5.09)	<0.001[d]
No	1				
Elixhauser Comorbidity Index					
0-4	1				
≥ 5	0.79	(0.63	-	0.98)	0.03[d]
Mental Health Diagnosis					
None	1				
>1	1.20	(1.12	-	1.31)	<0.001[d]
Median Residential Income[b]					
Quartile 4	1				
Quartile 3	0.87	(0.80	-	0.94)	<0.001[d]
Quartile 2	0.82	(0.76	-	0.89)	<0.001[d]
Quartile 1	0.70	(0.63	-	0.77)	<0.001[d]

© 2021 Huynh KA et al. *JAMA Network Open.*

eTable 8. Results of the Multivariable Logistic Regression Analysis Examining the Probability of Autologous Breast Reconstruction (continue)

	OR	(95% Confidence Interval)			P-value
Insurance Payer[c]					
Private	1				
Medicare	0.61	(0.54	-	0.69)	<0.001[d]
Medicaid	0.65	(0.59	-	0.72)	<0.001[d]
Self-pay	0.60	(0.47	-	0.76)	<0.001[d]
Other	0.88	(0.74	-	1.05)	0.16
Interaction Term (Expansion)					
White	0.87	(0.76	-	1.00)	0.06
Black	0.63	(0.49	-	0.79)	<0.001[d]
Hispanic	0.53	(0.39	-	0.71)	<0.001[d]
Other Minorities	0.74	(0.59	-	0.95)	0.01[d]

Abbreviations: OR, odds ratio.
[a] *Other Minorities includes Asians, Native Americans, and Other Minority groups*
[b] *U.S. dollar ranges for quartiles vary by year in NIS (eTable 4).*
[c] *Private insurance category includes Blue Cross, commercial carriers, private Health Maintenance Organizations, and Preferred Provider Organizations. Other insurance category includes worker's compensation, CHAMPUS, CHAMPVA, Title V of the McKinney-Vento Homeless Assistance Act, and other government programs.*
[d] *Indicates a significant difference at 95% confidence interval.*

© 2021 Huynh KA et al. *JAMA Network Open.*
